# Genome-wide detection of genotype environment interactions for flowering time in *Brassica napus*

**DOI:** 10.3389/fpls.2022.1065766

**Published:** 2022-11-21

**Authors:** Xu Han, Qingqing Tang, Liping Xu, Zhilin Guan, Jinxing Tu, Bin Yi, Kede Liu, Xuan Yao, Shaoping Lu, Liang Guo

**Affiliations:** ^1^ National Key Laboratory of Crop Genetic Improvement, Huazhong Agricultural University, Wuhan, China; ^2^ Hubei Hongshan Laboratory, Wuhan, China

**Keywords:** *Brassica napus*, flowering time, QTN-by-environment interactions, multiple genome-wide association studies, differentially expressed gene, climatic index

## Abstract

Flowering time is strongly related to the environment, while the genotype-by-environment interaction study for flowering time is lacking in *Brassica napus*. Here, a total of 11,700,689 single nucleotide polymorphisms in 490 *B. napus* accessions were used to associate with the flowering time and related climatic index in eight environments using a compressed variance-component mixed model, 3VmrMLM. As a result, 19 stable main-effect quantitative trait nucleotides (QTNs) and 32 QTN-by-environment interactions (QEIs) for flowering time were detected. Four windows of daily average temperature and precipitation were found to be climatic factors highly correlated with flowering time. Ten main-effect QTNs were found to be associated with these flowering-time-related climatic indexes. Using differentially expressed gene (DEG) analysis in semi-winter and spring oilseed rapes, 5,850 and 5,511 DEGs were found to be significantly expressed before and after vernalization. Twelve and 14 DEGs, including 7 and 9 known homologs in *Arabidopsis*, were found to be candidate genes for stable QTNs and QEIs for flowering time, respectively. Five DEGs were found to be candidate genes for main-effect QTNs for flowering-time-related climatic index. These candidate genes, such as *BnaFLC*s, *BnaFT*s, *BnaA02.VIN3*, and *BnaC09.PRR7*, were further validated by the haplotype, selective sweep, and co-expression networks analysis. The candidate genes identified in this study will be helpful to breed *B. napus* varieties adapted to particular environments with optimized flowering time.

## Introduction

As the world’s most important oilseed crop, planting of *Brassica napus* spans a wide range of growth periods and climate zones (Yang et al., 2014). To meet the needs of adaptation, *B*. *napus* adjusts the correct time to flower. Flowering time determines the transition from the vegetative to the reproductive phase, and therefore, the nutrients are available for remobilization at seed filling (Han et al., 2021). Early flowering facilitates mechanical harvesting and rotation with other crops, whereas late flowering enhances stem development, thus improving lodging resistance (Cui et al., 2021). Although previous studies have revealed the genetic basis of flowering time in *B*. *napus*, no studies have been reported on the genetic dissection of flowering time plasticity, namely, genotype-by-environment interaction (G by E).

The genetic basis of flowering time has been well-studied in the model plant *Arabidopsis thaliana* (Mouradov et al., 2002; Putterill et al., 2004; Bouché et al., 2016). The genetic networks underlying flowering consist of six major pathways interconnected, namely, photoperiod, vernalization, gibberellin, autonomous, thermal clock, and aging pathways (Putterill et al., 2004). Epigenetic regulation, miRNAs, phytohormones, sugar status, and signaling also play important roles in flowering time control (Bouché et al., 2016). In *B. napus*, the polyploid nature of *B. napus* has resulted in flowering-time-related genes undergoing extensive subfunctionalization (Schiessl, 2020). It has been demonstrated that there is a sophisticated network of interactions among *FLOWERING LOCUS C* homologs with different expression patterns in organs and development stages (Zou et al., 2012). *LOWERING LOCUS T* and *TERMINAL FLOWER 1* were found to have pleiotropic effects on flowering time, despite their redundancy in *B. napus* genome (Guo et al., 2014). Therefore, it demands more genetic basis research on flowering time in *B*. *napus*.

Flowering time is strongly influenced by the environment. A decrease in day length delays flowering in *B*. *napus*. A period of cooler temperature will determine vernalization and ensure reproductive development (Matar et al., 2021). Precipitation has been reported to have different effects on flowering phenology in different species (Zhang et al., 2018). Many genes have been reported to influence flowering time in response to the environment. *FLOWERING LOCUS T* (FT) was found to induce flowering through long-distance signaling by activating seasonal changes in day length (Corbesier et al., 2007). The epigenetic silencing of *FLC* accelerates flowering by prolonged cold vernalization (Bastow et al., 2004). H2A.Z incorporates *BraA.FT.a* chromatin at high ambient temperature and delays flowering time in *B. rapa* (Del Olmo et al., 2019). In *B. napus*, *Cycling Dof Factor1* delays the flowering time and was induced in response to low temperature (Xu and Dai, 2016). *BnNAC485* altered flowering time in response to abiotic stress (Ying et al., 2014). However, *B. napus* has developed two eco-types in China, namely, semi-winter oilseed rapes (SWORs) and spring oilseed rapes (SORs), to adapt different geographical environments and climates, leading to more complex molecular mechanisms of flowering time (Song et al., 2020).

In response to climate change, G by E is of fundamental importance in plant breeding and adaptation (Arnold et al., 2019; Zhao et al., 2022). In *B. napus*, the G by E of seed yield and oil content were found to exert specific adaptation to climates (Zhang et al., 2013a). Genotype and temperature interactions of seed oil content were found to be differential at the level of gene expression profiles (Zhu et al., 2012). Moreover, quantitative and population genetics have shown great power to bridge the gap between genomic diversity and phenotypic plasticity (Wu, 1998; Kusmec et al., 2017; Liu et al., 2021). For G by E studies on flowering time, four environmentally sensitive quantitative trait loci for flowering time identified in 473 *Arabidopsis* accessions were found to be related to adaptation (Li et al., 2010). It has been found that interacting flowering-time-related genes differentially respond to the temperature at the early growth stage in rice (Guo et al., 2020). Quantitative trait nucleotide (QTN)-by-environment interaction (QEI) mapping for flowering time has been performed in a doubled haploid *B. napus* population (Shen et al., 2018). Although many genome-wide association studies (GWAS) for flowering time have been reported in *B*. *napus* (Xu et al., 2016; Song et al., 2020; Helal et al., 2021; Hu et al., 2022), knowledge about QEI for flowering time detected by GWAS is scarce.

Recently, the newly published method 3VmrMLM provides a solution for QEI detection in GWAS (Li et al., 2022a). Here, we investigated the landscape of flowering time plasticity of 490 *B. napus* accessions in eight environments. A total of 11,700,689 single nucleotide polymorphisms (SNPs) were used to detect main-effect QTNs for flowering time and related climatic index and QEIs for flowering time. The transcriptome of SWORs and SORs before and after vernalization was used to identify the candidate genes around QTNs and QEIs. Co-expression, haplotype, and selection sweep analysis were used to further validate the candidate flowering time genes in specific eco-oilseed rapes. Our finding will facilitate the breeding for adaptation to particular environments with optimized flowering time in *B. napus*.

## Materials and methods

### Germplasm, phenotypic, and genomic data

A diversity panel of 490 *B. napus* accessions collected from Xu et al. (2016) was used in this study. This panel was cultivated in eight natural environments, i.e., Wuhan 2013 and 2014 (WH2013 and WH2014), Changsha 2013 and 2014 (CS2013 and CS2014), Nanjing 2013 and 2014 (NJ2013 and NJ2014), Ezhou 2013 (EZ2013), and Chongqing 2013 (CQ2013). Additionally, the Gangan and ZS11 cultivars for RNA-seq were planted in Wuhan 2018 at the experimental stations of Huazhong Agricultural University. The design of field trial of the above materials and the acquisition of phenotypic data were the same as those used in the previous study (Xu et al., 2016). The re-sequencing genome data were obtained from Tang et al. (2021). The *B*. *napus* genome (*B. napus* ZS11 v0) from BnPIR (Song et al., 2020; Song et al., 2021) (http://cbi.hzau.edu.cn/bnapus/index.php) was used as the reference genome.

### Statistical analysis for phenotypic data

By using the “lme4” R package (Bates et al., 2015), the best linear unbiased prediction (BLUP) model was fitted to each *B. napus* accession:


Phenotype ~ (1|Accession) + (1|Environment)

Taking into account the variations between eight environments as phenotypic variance derived from environmental factors, broad-sense heritability ( $h_{B}^{2}$ ) was estimated using the following equation by treating populations as a random effect and the environments as an environment effect, where $\sigma_{g}^{2}$ and $\sigma_{e}^{2}$ is the variance derived from genetic and environmental effects, respectively (Knapp et al., 1985).


$$h_{B}^{2}=\frac{\sigma_{g}^{2}}{\sigma_{g}^{2}+\sigma_{e}^{2}}$$

### Identification of flowering-time-related climatic index

Climatic data for daily average temperature (TAVG, °F) and precipitation (PRCP, in) were retrieved from the National Oceanic and Atmospheric Administration (https://www.noaa.gov/weather). Due to the lack of climatic data for Ezhou, there were climatic datasets of seven environments in total, i.e., WH2013 and WH2014 (114.05°E, 30.60°N; Station ID: GHCND: CHM00057494), CS2013 and CS2014 (112.87°E, 28.23°N; GHCND: CHM00057687), NJ2013 and NJ2014 (118.90°E, 31.93°N; GHCND: CHM00058238), and CQ2013 (106.48°E, 29.58°N; GHCND: CHM00057516). Climatic data were obtained from the day after being planted to the 200 days after planting (DAP). For each window from a starting day (3 DAP) to an end day (41 DAP) during *B*. *napus* growth, the average value of the climatic index and their correlation with the environmental mean vector for flowering time was calculated by CERIS analytical package (Li et al., 2021; https://github.com/jmyu/CERIS_JGRA). The most relevant climatic index for flowering time was chosen according to the highest correlation between environmental means and climatic index with corresponding window. Reaction norms were calculated as described in Guo et al. (2020) and Liu et al. (2020), using environmental mean and environmental climatic index as x-axis and phenotype as y-axis. Each line represented an individual and was shown by fitted linear regression. The intercept and slope were used to perform GWAS further.

### Detecting QTNs and QEIs by GWAS

The intersection of the accessions in phenotypic and genotypic datasets, i.e., 490 accessions with 11,700,689 SNPs, were used for GWAS using 3VmrMLM (Li et al., 2022a) *via* software IIIVmrMLM (Li et al., 2022b). Flowering time QTNs were obtained from separate analyses of phenotypic data from eight environments and joint environmental analyses of these datasets. The reaction norms between flowering time and climatic index were also used to conduct GWAS by 3VmrMLM. QEIs for flowering time were obtained by joint environment analyses of the above phenotypic datasets in eight environments. Population structure and kinship matrix were considered in 3VmrMLM analysis, and the “svpal” parameter was set as 0.01. According to Tang et al. (2021), the population structure calculated as *K*=3 was used in the analysis. The threshold was set at 0.05/*m* for significant QTNs and QEIs and LOD score ≥ 3.0 for suggested QTNs and QEIs, where *m* is the number of markers (Li et al., 2022a; Li et al., 2022b). According to the LD interval estimated by Tang et al. (2021), stable QTNs were defined as QTNs identified in at least three environments within the 100-kb upstream and downstream regions.

### Identification of candidate genes

To identify candidate genes for flowering-time-related QTNs and QEIs, genes within the 100 kb upstream and downstream regions of each QTN or QEI were extracted according to the LD interval estimated by Tang et al. (2021). Then, two strategies were employed. First, the *B. napus* homologs of *Arabidopsis* flowering time genes downloaded from FLOR-ID (http://www.flor-id.org) were selected and considered as known genes. Second, new candidate genes were identified using differentially expressed genes (DEGs) in two SWORs (Gangan and ZS11) before and after vernalization and in two SORs (Westar and No. 2127). The *t*-test was adopted in the hypothesis testing for haplotype analysis; *p*< 0.05, *p*< 0.01, and *p*< 0.001 indicated the significances at 0.05, 0.01, and 0.001 probability levels, respectively.

### Differential expression analysis based on RNA-seq

The leaves of Westar, No. 2127, Gangan, ZS11 at 24 and 147 DAP were collected for RNA-seq with two biological replicates. Total RNA was extracted using the TIANGEN RNAprep Pure Plant Kit. Sequencing libraries were generated using the NEBNext^®^ UltraTM RNA Library Prep Kit for Illumina^®^ (NEB, USA) and were sequenced on an Illumina Hiseq 4000 platform. The detailed processes were described in Tan et al. (2022). We used MultiQC (Ewels et al., 2016) to perform quality control and Salmon (Patro et al., 2017) to quantify the RNA-seq reads of annotated genes in the reference ZS11. DESeq2 was used for differential expression analysis (Love et al., 2014). The threshold for DEG is set as the absolute value of log_2_FoldChange >1 and adjusted *p<* 0.05 (two-tailed Student’s *t*-test; Tan et al., 2022).

### Identification of selective sweep signals

To detect the regions under selective sweeps between SWOR and SOR, XP-CLR (v1.1.1), a genome scan using the composite likelihood approach was performed in sub-populations (Chen et al., 2010). Each chromosome was analyzed using the XP-CLR command with the parameters “–ld 0.99 –phased –maxsnps 200 –size 100000 –step 10000.” Non-overlapping 20-kp windows within the top 20% XP-CLR scores were merged into one single region, and then, these regions in the top 1% of XP-CLR scores were considered as candidate selective regions (An et al., 2019).

### Construction of co-expression network

According to the above RNA-seq datasets, Pearson correlation analysis was calculated between candidate genes and DEGs in SWORs and SORs, respectively. Significant genes were considered to be co-expressed when Pearson correlation coefficient was >0.80 and *p*-value was<0.05. Network visualization was implemented with the Cytoscape package (Shannon et al., 2003).

## Results

### Flowering time plasticity and related climatic index for *B. napus*

**Figure 1:**
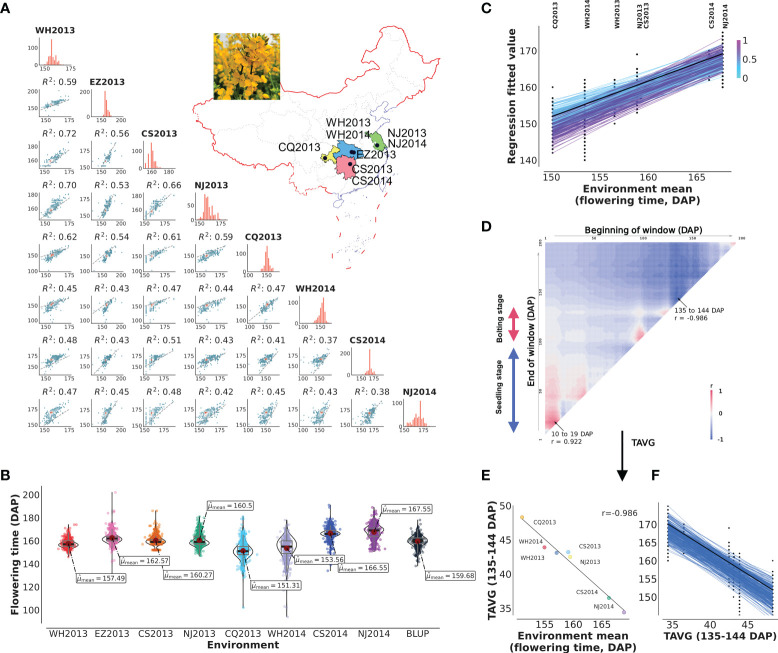
Plasticity of flowering and reaction norm of its associated window to daily average temperature (TAVG). **(A, B)** Characteristics and pairwise correlations of flowering time of 490 *B napus* in eight environments. WH2013, Wuhan in 2013; WH2014, Wuhan in 2014; CS2013, Changsha in 2013; CS2014, Changsha in 2014; NJ2013, Nanjing in 2013; NJ2014, Nanjing in 2014; CQ2013, Chongqing in 2013; EZ2013, Ezhou in 2013; BLUP, the best linear unbiased prediction value. **(C)** Reaction norm for flowering time based on a numerical order of environmental mean. Dots are the observed flowering time phenotypic values. The line with black color represents the ZS11 cultivar. The color of the line represents the value of the slope. **(D)** Search for the window to TAVG, which is highly correlated with environmental mean of flowering time (from planting to 200 days after planting, DAP). TAVG within the window of 10–19 and 135–144 DAP was chosen and denoted as TAVG_10–19_ and TAVG_135–144_. **(E, F)** Significant correlation and reaction norm between TAVG_135–144_ and environmental mean of flowering time.

Complex flowering time variation was observed in diversity group of 490 *B. napus* oilseed rapes, including 49 SORs, 20 winter oilseed rapes, 326 SWORs, and 95 mixed type oilseed rapes, grown in eight natural environments (**Figure 1A**; **Supplementary Table S1**). The means plus standard deviations of the eight environments WH2013, WH2014, CS2013, CS2014, NJ2013, NJ2014, CQ2013, EZ2013, and BLUP values were 155.49 ± 3.80, 153.56 ± 9.61, 160.27 ± 4.29, 166.55 ± 5.44, 160.50 ± 5.49, 167.55 ± 6.38, 151.31 ± 7.82, 162.57 ± 5.30, and 159.68 ± 4.83 (DAP), respectively (**Figure 1B**). The correlation of each pair of environments ranged from 0.37 to 0.72 (0.50 ± 0.09). The coefficients of variation, skewness, and kurtosis of the trait in eight environments illustrated that flowering time is a typical quantitative trait (**Supplementary Table S2**). The broad-sense heritability for flowering time is 0.86. More importantly, joint regression analysis modeled with environmental mean showed the presence of a significant phenotypic plasticity (**Figure 1C**).

Climate change is altering the environment in which all plants grow. To understand the effect of climatic index on flowering time plasticity, the correlation between environmental means and climatic index (TAVG and PRCP) for different growth windows was predicted by CERIS (**Supplementary Table S3**). The results of the correlation pattern between TAVG and flowering time showed a positive correlation at early seedling stage and a negative trend after bolting stage, while the pattern of PRCP was exactly opposite (**Figure 1D**; **Supplementary Figure S1A**; **Supplementary Table S4**). The windows with the highest negative (TAVG_135–144_ and PRCP_3–41_) and positive correlations (TAVG_10–19_ and PRCP_133–169_) were chosen as the most related climatic index for further analysis (**Figures 1E** **, F**; **Supplementary Figures S2A** **–F**; **Supplementary Table S4**). TAVG_135–144_ (r = −0.986) showed higher correlation with flowering time than PRCP_3–41_ (r = −0.809). TAVG_10–19_ (r = 0.922) showed higher correlation with flowering time than PRCP_133–169_ (r = 0.901). It is noted that these windows are surrounded by other windows with slightly decreasing correlation values (**Figure 1D**; **Supplementary Figure S1**).

### Detection of QTNs for flowering time

**Figure 2:**
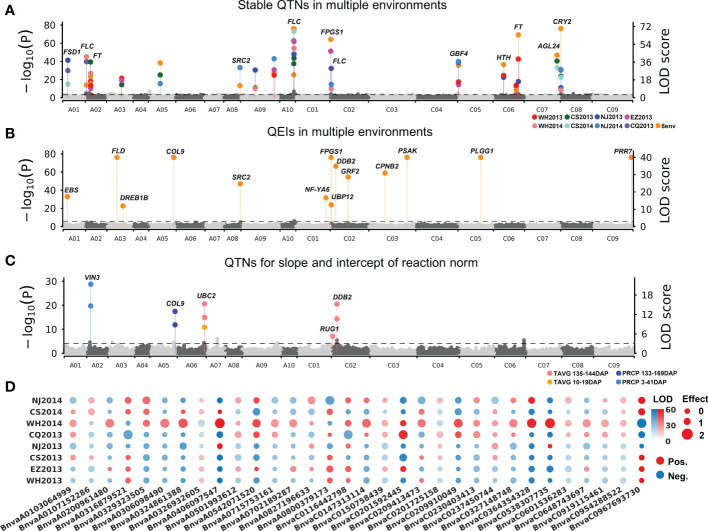
Manhattan plots for flowering time of 490 *B napus* accessions. **(A)** Nineteen stable main-effect QTNs and their candidate genes for flowering time in eight single environment analyses and multiple environments joint analysis. **(B)** QTN-by-environment interactions (QEIs) and their candidate genes for flowering time in multiple environments joint analysis. **(C)** Ten main-effect QTNs for slope and intercept of reaction norm for flowering-time-related climatic indexes. **(D)** Additive-by-environment interaction effects of 32 QEIs in eight environments. The size of dot: absolute value of additive-by-environment interaction effect. Red/blue dot: positive/blue value. WH2013, Wuhan in 2013; WH2014, Wuhan in 2014; CS2013, Changsha in 2013; CS2014, Changsha in 2014; NJ2013, Nanjing in 2013; NJ2014, Nanjing in 2014; CQ2013, Chongqing in 2013; EZ2013, Ezhou in 2013.

To detect QTNs for flowering time, the phenotypes in each of the eight environments were used to associate with 11,700,689 SNPs using 3VmrMLM under population structure and polygenic background control. As a result, 55, 57, 42, 49, 54, 50, 44, and 43 significant QTNs at the critical *p*-value of 4.27e−09 (=0.05/*m*, where *m* is the number of markers) and 10, 5, 14, 10, 8, 13, 13, and 13 suggested QTNs (with the LOD score ≥ 3.0 but the *p* > 0.05/*m*) were identified for WH2013, WH2014, CS2013, CS2014, NJ2013, NJ2014, CQ2013, and EZ2013, respectively (**Supplementary Table S5**; **Supplementary Figure S3**). In addition, flowering phenotypes from eight environments were used to perform joint analysis by 3VmrMLM. Sixty-eight significant and 11 suggested QTNs were identified. Based on the above QTNs in single and multiple environments analyses, 19 stable QTNs were identified in at least three environments (**Figure 2A**; **Table 1**).

**Table 1:** Nineteen stable QTNs for *B. napus* flowering time and their candidate genes.

Genome-wide association studies						Comparative genomics analysis			
Chr	Pos (bp)	Marker	LOD	*R* ^2^	Environments^a^	Gene ID	Abbr.	Function	Reference
A10	24056113–24056153	*BnvaA1024056153, BnvaA1024056113, BnvaA1024056139*	39.96–117.88	0.49–2.10	E1, E3, E4, E5, E6, E7, E9				
C08	912878	*BnvaC0800912878*	7.94–82.49	0.53–2.42	E1, E3, E4, E5, E6, E7, E8	*BnaC08G0010300ZS*	*CRY2*	Cryptochrome-2	Sharma et al., 2022
C05	1376324	*BnvaC0501376324*	13.31–36.7	0.12–0.81	E1, E2, E6, E7, E9	*BnaC05G0024000ZS*	*GBF4*	G-BOX BINDING FACTOR 4	
A09	56413085–56417605	*BnvaA0956413085, BnvaA0956414961, BnvaA0956417605*	22.79–39.42	0.14–1.43	E1, E2, E9, E7				
A10	23668965–23770033	*BnvaA1023770033, BnvaA1023668965*	23.22–84.79	0.11–2.11	E1, E2, E4, E8	*BnaA10G0244800ZS*	*FLC*	MADS-box protein FLOWERING LOCUS C	Tadege et al., 2001
A02	9020851–9105883	*BnvaA0209020851, BnvaA0209054089, BnvaA0209105883*	9.42–24.67	0.08–1.45	E1, E2, E3, E9	*BnaA02G0156900ZS*	*FT*	Protein FLOWERING LOCUS T	Wang et al., 2009
C02	2400090–2502621	*BnvaC0202402020, BnvaC0202400090, BnvaC0202502621, BnvaC0202402023*	8.82–29.63	0.30–1.47	E3, E5, E6, E7	*BnaC02G0039100ZS*	*FLC*	MADS-box protein FLOWERING LOCUS C	Tadege et al., 2001
C07	55454986–55455005	*BnvaC0755455005, BnvaC0755454986*	30.53–43.11	0.14–0.67	E1, E4, E5	*BnaC07G0458500ZS*	*AGL24*	MADS-box protein AGL24	Yu et al., 2002
C02	1592445	*BnvaC0201592445*	47.10–59.13	0.19–1.06	E1, E9	*BnaC02G0022200ZS*	*FPGS1*	Folylpolyglutamate synthase	
A01	8566494–8643230	*BnvaA0108643230, BnvaA0108602009, BnvaA0108566494*	13.70–37.97	0.55–1.29	E5, E6, E8	*BnaA01G0146300ZS*	*FSD1*	Fe superoxide dismutase. Superoxide dismutase	
A05	19689622	*BnvaA0519689622*	14.27–35.31	0.13–0.48	E1, E4, E7				
A08	27196633–27207043	*BnvaA0827196633, BnvaA0827207043, BnvaA0827196973*	12.21–30.51	0.13–1.71	E1, E7	*BnaA08G0296600ZS*	*SRC2*	soybean gene regulated by cold-2	
A02	8776765–8833814	*BnvaA0208833814, BnvaA0208776765*	16.66–36.12	0.24–0.76	E1, E2, E4				
A03	25426194–25520626	*BnvaA0325426194, BnvaA0325520626*	13.14–19.74	0.37–0.63	E2, E4, E9				
C06	17894906	*BnvaC0617894906*	20.65–33.50	0.15–0.41	E1, E2, E6				
C06	39070745–39079766	*BnvaC0639079766, BnvaC0639070745*	7.60–12.55	0.08–0.55	E1, E2, E4	*BnaC06G0286700ZS*	*HTH*	Omega-Hydroxy Fatty Acyl Dehydrogenase	
C06	42697888	*BnvaC0642697888*	16.35–63.64	0.31–0.74	E1, E2, E6	*BnaC06G0323800ZS*	*FT*	Protein FLOWERING LOCUS T	Wang et al., 2009
A02	1946991–2001373	*BnvaA0201946991, BnvaA0202001373, BnvaA0202001103*	4.53–41.57	0.07–1.03	E1, E3, E8	*BnaA02G0035100ZS*	*FLC*	MADS-box protein FLOWERING LOCUS C	Tadege et al., 2001
A09	24518838–24519761	*BnvaA0924518838, BnvaA0924519761*	9.29–27.99	0.24–0.65	E4, E3, E6				

a E1: multi-environments joint GWAS; E2: WH2013; E3: WH2014; E4: CS2013; E5: CS2014; E6: NJ2013; E7: NJ2014; E8: CQ2013; E9: EZ2013.

### Detection of QTN-by-environment interactions for flowering time in multiple environments

All the datasets in eight environments were used to conduct joint analysis for identifying QEIs using 3VmrMLM. As a result, 32 significant QEIs and 4 suggested QEIs were identified, including 10 significant QEIs overlapped with the above stable QTNs (**Supplementary Table S6**). Among these significant QEIs, 20 were found to have the highest absolute value of additive-by-environment interaction effects in WH2014 than those in other environments (**Figures 2B, D**), e.g., BnvaC0967693730 has an additive-by-environment interaction effect of −1.85 in WH2014 than those in other environments (**Supplementary Table S6**; LOD = 67.17; *R* ^2^ = 1.07%). The two loci BnvaC0967693730 and BnvaA0406097547 have the highest *R* ^2^ (LOD = 67.17; *R^2^* = 1.07% and LOD = 66.42; *R* ^2^ = 1.06%, respectively).

### Detection of QTNs for flowering-time-related climatic index

To obtain reaction norms of flowering-time-related climatic index, joint regression analyses were performed on phenotypes and the above flowering-time-related climatic indexes (TAVG_135–144_, PRCP_3–41_, TAVG_10–19_, and PRCP_133–169_; **Supplementary Table S4**). The intercept and slope of reaction-norm parameters were used to detect QTNs for flowering-time-related climatic indexes using 3VmrMLM. As a result, 10 QTNs for reaction norm parameters of *B. napu*s flowering time were commonly identified with the above stable QTNs or QEIs, including 5, 2, 1, and 2 for TAVG_135–144_, PRCP_3–41_, TAVG_10–19_, and PRCP_133–169,_ respectively (**Figure 2C**; **Supplementary Table S7**).

### Prediction of candidate genes for flowering time

**Figure 3:**
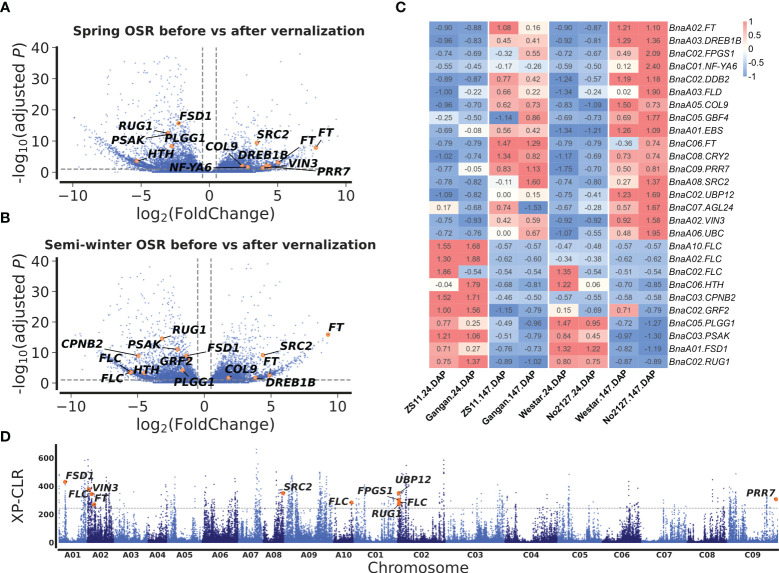
Differentially expressed gene (DEG) analysis before and after vernalization and selection sweeps between semi-winter and spring oilseed rapes (SWORs and SORs). Volcano plots of DEGs in SORs **(A)** and SWORs **(B)**. The y-axis is the adjusted *p*-value and the x-axis is log_2_ fold-change (FC) before and after vernalization. Gray lines are at the absolute value of log_2_FC = 1 or adjusted *p*-value = 0.05. **(C)** The expression profiling of 27 candidate genes around main-effect QTNs and QEIs for flowering time in two SWORs and two SORs in the 2018–2019 growing season in Wuhan. DAP, days after planting. **(D)** Selective sweeps between SWORs and SORs by XP-CLR. The horizontal dashed lines indicate the cutoff in the top 1% of XP-CLR scores. Candidate genes for flowering time are marked above the selective sweep peaks.

To mine candidate genes among the above QTNs and QEIs, DEGs analysis was conducted before and after vernalization. A total of 5,511 DEGs were identified in two SORs before and after vernalization (**Figure 3A**; **Supplementary Table S8**), and 5,850 DEGs were identified in two SWORs before and after vernalization (**Figure 3A**; **Supplementary Table S9**). Then, according to *Arabidopsis* gene annotation, 12 candidate genes were found to be associated with flowering time in approximately above 19 stable QTNs, including 7 known flowering-time-related homologs in *Arabidopsis* and 5 newly discovered genes (**Table 1**). Using the same methods, 14 candidate genes were identified to be located in the above 32 QEIs, including 9 homologs of known genes, in which their homologs are related to flowering time and environments in *Arabidopsis* and 5 newly identified genes (**Table 2**). In addition, five candidate genes were found to be associated with flowering-time-related climatic index, including two genes (*BnaC02.DDB2* and *BnaA05.COL9*) commonly identified in QEIs and three genes (*BnaA02.VIN3*, *BnaC02.RUG1*, and *BnaA06.UBC2*) commonly found to be associated with the flowering time QTNs (**Supplementary Table S7**).

**Table 2:** Fourteen candidate genes for *B. napus* flowering time around significant QTN-by-environment interactions.

Genome-wide association studies					Comparative genomics analysis				Evidences for environmental interaction	
Chr	Pos (bp)	Marker	LOD	*R* ^2^ (%)	Gene ID	Abbr.	Function	Reference	Environment	Differences of flowering time under various environments
C09	67693730	*BnvaC0967693730*	67.17	1.07	*BnaC09G0614800ZS*	*PRR7*	Two-component response regulator-like APRR7	Nakamichi et al., 2007	Circadian clock	*prr7* single mutant is late flowering under LD conditions
C05	38307735	*BnvaC0538307735*	62.47	1.01	*BnaC05G0345200ZS*	*PLGG1*	Plastidal glycolate/glycerate translocator			
C03	64354328	*BnvaC0364354328*	60.84	0.97	*BnaC03G0665500ZS*	*PSAK*	Photosystem I reaction center subunit K			
C02	1592445	*BnvaC0201592445*	58.92	0.94	*BnaC02G0022200ZS*	*FPGS1*	Folylpolyglutamate synthase			
A05	42071520	*BnvaA0542071520*	50.18	0.79	*BnaA05G0456200ZS*	*COL9*	Zinc finger protein CONSTANS-LIKE 9	Cheng and Wang, 2005	Circadian clock	*col9* single mutant is early flowering under LD conditions
A03	16879521	*BnvaA0316879521*	42.43	0.70	*BnaA03G0318500ZS*	*FLD*	FOLOWERING LOCUS D	Zhang et al., 2013b	Circadian clock	*fld* single mutant is late flowering under both SD and LD conditions
C02	9413473	*BnvaC0209413473*	34.92	0.55	*BnaC02G0132800ZS*	*DDB2*	Damaged DNA-binding proteins 2 required for UV-B tolerance	Al Khateeb and Schroeder, 2007	Light signaling	ddb2 suppressed the early flowering time of *det1* under long-day conditions
C03	27148748	*BnvaC0327148748*	30.92	0.48	*BnaC03G0400500ZS*	*CPNB2*	Chaperonin 60 subunit beta			
C02	30403413	*BnvaC0230403413*	28.66	0.45	*BnaC02G0311500ZS*	*GRF2*	G-box binding factor GF14 omega encoding a 14-3-3 protein	Liu et al., 2012	Unclear	*BnGRF2a* transgenic lines delays flowering
A08	27196633	*BnvaA0827196633*	24.73	0.38	*BnaA08G0296600ZS*	*SRC2*	Involved in Protein Storage Vacuole targeting.			
A01	7152286	*BnvaA0107152286*	17.37	0.27	*BnaA01G0121900ZS*	*EBS*	PHD finger family protein	López-González et al., 2014	Epigenetic regulation	*ebs* mutants repressed flowering
C01	50758439	*BnvaC0150758439*	16.63	0.27	*BnaC01G0442400ZS*	*NF-YA6*	Nuclear factor Y, subunit A6	Siriwardana et al., 2016	Photoperiod	*NF-YA* can be positive regulators of photoperiod dependent flowering
C02	1725158	*BnvaC0201725158*	12.62	0.19	*BnaC02G0024600ZS*	*UBP12*	Ubiquitin carboxyl-terminal hydrolase 12	Cui et al., 2013	Circadian clock	ubp12 single mutant is slightly early flowering under both SD and LD conditions
A03	26932605	*BnvaA0326932605*	11.96	0.18	*BnaA03G0486700ZS*	*DREB1B*	Dehydration-responsive element-binding protein 1B	Seo et al., 2009	Cold	Response to ABA treatment

Among these candidate genes, *BnaFT*s, *BnaA05.COL9*, *BnaA08.SRC2*, and *BnaA03.DREB1B* were significantly upregulated before vernalization in both SWORs and spring SORs, while *BnaFLCs*, *BnaA01.FSD1*, *BnaC02.RUG1*, *BnaC05.PLGG*, and *BnaC03.PSAK* were significantly upregulated after vernalization (**Figures 3A** **, B**). Interestingly, *BnaA02.VIN3* and *BnaC02.FPGS* were only significantly upregulated before vernalization in SOR, which may indicate different functions between eco-types.

### Validation of candidate genes

**Figure 4:**
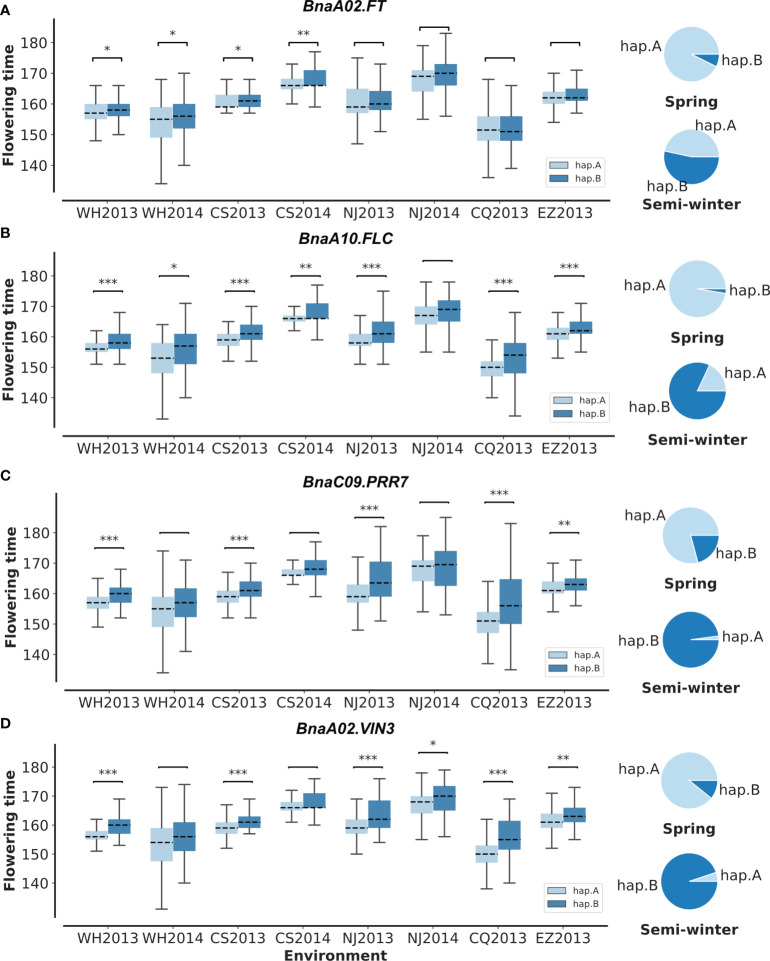
Haplotype analysis of *BnaC02.FT*, *BnaA10.FLC*, *BnaC09.PRR7*, and *BnaA02.VIN3* **(A–D)**. In the boxplot, significant differences for flowering time between each haplotype are calculated in eight environments with *t*-test. In pie plots, the haplotype frequencies of each gene in semi-winter and spring oilseed rapes are marked. WH2013, Wuhan in 2013; WH2014, Wuhan in 2014; CS2013, Changsha in 2013; CS2014, Changsha in 2014; NJ2013, Nanjing in 2013; NJ2014, Nanjing in 2014; CQ2013, Chongqing in 2013; EZ2013, Ezhou in 2013. *p = 0.05, **p = 0.01, and ***p = 0.001.

**Figure 5:**
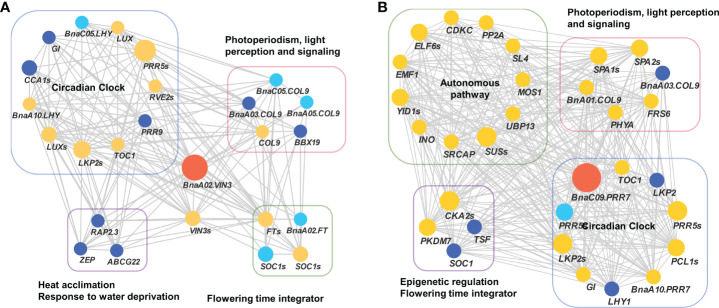
Co-expression network of *BnaVIN3* and *BnaPRR7* with co-expressed genes related to flowering time **(A, B)**. Light blue, dark blue, and yellow node indicate co-expressed genes that were detected in semi-winter, spring, and both types of oilseed rapes, respectively. The size of each node represents the number of genes in each gene family.

To validate the above flowering time candidate genes, we conducted selective sweep, haplotype, and co-expression analysis. First, by performing XP-CLR between SWORs and SORs, 954 selective sweeps were detected (**Supplementary Table S10**). Eleven candidate genes for flowering time were found in the selective sweep, e.g., *BnaFLCs*, *BnaFTs*, *BnaC02.FPGS1*, *BnaA08.SRC2*, *BnaA01.FSD1*, *BnaA02.VIN3*, and *BnaC09.PRR7*. Second, haplotype analyses were further conducted in these genes. For *BnaA02.FT*, *BnaA10.FLC*, *BnaA02.VIN3*, and *BnaC09.PRR7*, significant difference exists between each haplotype in different environments (**Figures 4A–D**; **Supplementary Figures S4A–D**). Interestingly, the haplotype for early flowering tends to exist in SORs, while the haplotype for late flowering prefers to exist in SWORs. Moreover, the co-expression networks of *BnaA02.VIN3* and *BnaC09.PRR7* have been constructed using DEGs in SWORs and SORs, respectively (**Figures 5A, B**). The co-expressed genes of *BnaA02.VIN3* mainly participated in the circadian clock, photoperiodism, light perception, and signaling. Eight genes are specific co-expressed in SORs, including *BnaA07.ZEP* and *BnaC09.ABCG22* in response to water deprivation. Five genes are specific co-expressed in SWORs. On the other hand, the co-expressed genes of *BnaC09.PRR7* mainly participated in the circadian clock and autonomous pathway. Five and one genes are specific co-expressed in SORs and SWORs, respectively. *BnaCKA2*s and *BnaPKDM7*s participated in epigenetic regulation.

## Discussion

Although flowering time is strongly related to the environment, G by E studies for flowering time are lacking in *B. napus*. The current study analyzed the G by E for flowering time in the following three aspects. First, four windows of flowering-time-related climatic index were identified (TAVG_135–144_, PRCP_3–41_, TAVG_10–19_, and PRCP_133–169_) by CERIS. Second, 19 stable QTNs and 32 QEIs were found to be significantly associated with flowering time of 490 *B. napus* accessions in eight environments, and 10 QTNs were found to be associated with flowering-time-related climatic index. Finally, based on DEGs and homology with *Arabidopsis*, 12, 14, and 5 candidate genes were found to be associated with stable QTNs, QEIs, and QTNs for flowering-time-related climatic index, respectively. These candidate genes were further validated by the haplotype, selective sweep, and co-expression network analysis.

### Flowering-time-related climatic index in *B. napus* whole growth stages

It is well-known that the flowering time regulation of *B. napus* is in response to day length or vernalization (Reeves and Coupland, 2000). This study calculated the correlations between two climatic factors, TAVG and PRCP, and flowering time in seven environments. TAVG correlated positively with flowering time in vernalization and negatively with flowering time after the seedling stage (**Figure 1D**). In a previous study, a reduction in autumn or winter chilling delays floral transition in *B. napus* (O’Neill et al., 2019). An elevated growth temperature is equally efficient in inducing the flowering of *Arabidopsis* (Balasubramanian et al., 2006). However, the transition or critical point of these two stages is unclear. For PRCP, this study reported the relationships between PRCP and flowering time in *B. napus* for the first time. Although the correlation coefficients are lower than TAVG, PRCP was found to be correlated negatively with flowering time in early development and positively later (**Supplementary Figure S1**). This result is consistent with a previous study in *Arabidopsis* that flowering time correlated negatively with fall and winter precipitations and positively with summer precipitation (Vidigal et al., 2016).

### Genetic basis for flowering time in *B. napus*

In this study, multi-environment joint GWAS improved the power on identifying more QTNs than single environment GWAS. We dissected the genetic basis for flowering time in the following three aspects. First, 12 flowering time candidate genes were mined in approximately 19 stable QTNs for flowering time. Seven genes are previously reported, e.g., *BnaFLC*s (Tadege et al., 2001), *BnaFTs* (Wang et al., 2009), *BnaAGL24* (Yu et al., 2002), and *BnaCRY2* (Sharma et al., 2022), whereas five genes are newly identified, which are differentially expressed before and after the vernalization of different ecotypes (**Figure 3**; **Supplementary Tables S8**, **S9**). Second, it is worth noting that this study focused on the mining of flowering time genes related to the environments. Fourteen candidate genes were identified around 32 QEIs, including 9 known flowering time genes related to environments. For example, *BnaCOL9* and *BnaUBP12* are regulated by the circadian clock in the photoperiod pathway (Cheng and Wang, 2005; Cui et al., 2013). *BnaFLD* is subjected to the direct regulation by brassinosteroids (Zhang et al., 2013b). It has been reported that the overexpression of *BnaDREB1B* not only delayed flowering but also responded to cold (Seo et al., 2009). *BnaEBS* functions in the chromatin-mediated repression of floral initiation by H3K4me3 (López-González et al., 2014). Finally, five genes were found to be associated with flowering-time-related climatic index. *BnaC02.DDB2* and *BnaA05.COL9* were commonly identified in QEIs, and *BnaA02.VIN3*, *BnaC02.RUG1*, and *BnaA06.UBC2* were commonly found to be associated with the main effect flowering time QTNs.

In this study, the missing heritability exists, in which the total phenotypic variance explained of QEIs and QTNs is much less than the estimated broad-sense heritability. This can be explained in several ways. First, the population is not enough to detect rare variants. Second, allelic heterogeneity may be the reason for this phenomenon. Lastly, epigenetic variation is likely to be a source of missing heritability (Brachi et al., 2011). Moreover, some candidate genes for stable QTNs, e.g., *BnaA02.FT* and *BnaA10.FLC*, were found to be related to environments but were not identified in QEIs (**Figure 4**). This result is explained by multiple facets, e.g., the difference in phenotypic data among environments, the diversity of population accessions, and the power of QEI detection. In the previous study, *COL9* and *FLD* have been reported to regulate *FT* and *FLC*, respectively (Cheng and Wang, 2005; Jiang et al., 2009). *BnA05.COL9* and *BnaA03.FLD* were found to be candidate genes for QEIs in this study. We hypothesized that QEI may be associated with direct environmental response upstream regulators due to the complexity of transcription and epigenetic regulations of flowering (Bouché et al., 2016).

### *BnaA02.VIN3* and *BnaC09.PRR7* are potential G by E genes for flowering time

In *Arabidopsis*, *VIN3* acts together with *PRC2* to repress histone marks at *FLC* in response to vernalization (Kim and Sung, 2013). *PRR7* was reported to coordinate with *PRR9* and *PRR5* and regulate flowering time through the canonical *CO*-dependent photoperiodic pathway (Nakamichi et al., 2007). In this study, *BnaA02.VIN3* and *BnaC09.PRR7* have been shown to be crucial G by E genes for flowering time. There are three pieces of evidence. First, *BnaA02.VIN3* is significantly associated with ChrA02-6152101 (LOD = 13.14) for flowering-time-related climatic factors and with ChrA02-6374324 (LOD = 12.17) for flowering time in WH2013. *BnaC09.PRR7* is significantly associated with the QEI, ChrC09-67693730 (LOD = 67.17), by multi-environment GWAS. Second, *BnaA02.VIN3* and *BnaC09.PRR7* are DEGs before and after vernalization and in the selective sweep between SORs and SWORs (**Figure 2**). Then, in these genes with significant haplotype differences, their haplotypes for early flowering tend to exist more in SORs (**Figures 4C** **, D**). Lastly, co-expression networks were constructed for *BnaA02.VIN3* and *BnaC09.PRR7*. Some relationships have been proven, e.g., *PRR7* with *LHY* (Liu et al., 2013), *PRR7* with *PRR5* (Nakamichi et al., 2007), and *VIN3* with *CCA1* and *LHY* (Kyung et al., 2022).

In summary, we dissected the G by E for flowering time for *B. napus* from different eco-types in eight environments. Four windows of flowering-time-related climatic index were identified. Stable QTNs and QEIs for flowering time and their candidate genes were identified. These findings provide valuable information that can be used to breed *B. napus* varieties with optimized flowering time by pyramiding favorable alleles. The candidate genes will also greatly promote the dissection of flowering time mechanisms in different eco-types.

## Data availability statement

The original contributions presented in the study are publicly available. This data can be found here: NGDC, PRJCA012445 and CRA008501.

## Author contributions

LG and XH conceived this study. QT, LX, ZG, JT, and BY performed the field experiments. XH performed the bioinformatics analysis and wrote the manuscript. JT, BY, KL, XY, SL, and LG revised the manuscript. All authors approved the submitted version.

## Funding

This work was supported by grants from the National Natural Science Foundation of China (U2102217), Key Research and Development Program of Hubei (2021ABA011) and Higher Education Discipline Innovation Project (B20051).

## Acknowledgments

We would like to thank Prof. Yuan-Ming Zhang (College of Plant Science and Technology, Huazhong Agricultural University, Wuhan) for improving the language within the manuscript.

## Conflict of interest

The authors declare that the research was conducted in the absence of any commercial or financial relationships that could be construed as a potential conflict of interest.

## Publisher’s note

All claims expressed in this article are solely those of the authors and do not necessarily represent those of their affiliated organizations, or those of the publisher, the editors and the reviewers. Any product that may be evaluated in this article, or claim that may be made by its manufacturer, is not guaranteed or endorsed by the publisher.

## References

[B1] Al Khateeb W. M. Schroeder D. F. (2007). DDB2, DDB1A and DET1 exhibit complex interactions during arabidopsis development. Genetics 176, 231–242. doi: 10.1534/genetics.107.070359 17409070 PMC1893029

[B2] An H. Qi X. Gaynor M. L. Hao Y. Gebken S. C. Mabry M. E.. (2019). Transcriptome and organellar sequencing highlights the complex origin and diversification of allotetraploid *Brassica napus* . Nat. Commun. 10, 2878. doi: 10.1038/s41467-019-10757-1 31253789 PMC6599199

[B3] Arnold P. A. Kruuk L. E. B. Nicotra A. B. (2019). How to analyse plant phenotypic plasticity in response to a changing climate. New Phytol. 222, 1235–1241. doi: 10.1111/nph.15656 30632169

[B4] Balasubramanian S. Sureshkumar S. Lempe J. Weigel D. (2006). Potent induction of *Arabidopsis thaliana* flowering by elevated growth temperature. PloS Genet. 2, e106. doi: 10.1371/journal.pgen.0020106 16839183 PMC1487179

[B5] Bastow R. Mylne J. S. Lister C. Lippman Z. Martienssen R. A. Dean C. (2004). Vernalization requires epigenetic silencing of *FLC* by histone methylation. Nature 427, 164–167. doi: 10.1038/nature02269 14712277

[B6] Bates D. Mächler M. Bolker B. M. Walker S. C. (2015). Fitting linear mixed-effects models using lme4. J. Stat. Software 67, 1–48. doi: 10.18637/jss.v067.i01

[B7] Bouché F. Lobet G. Tocquin P. Périlleux C. (2016). FLOR-ID: an interactive database of flowering-time gene networks in *Arabidopsis thaliana* . Nucleic Acids Res. 44, D1167–D1171. doi: 10.1093/nar/gkv1054 26476447 PMC4702789

[B8] Brachi B. Morris G. P. Borevitz J. O. (2011). Genome-wide association studies in plants: The missing heritability is in the field. Genome Biol. 12, 232. doi: 10.1186/gb-2011-12-10-232 22035733 PMC3333769

[B9] Cheng X. F. Wang Z. Y. (2005). Overexpression of *COL9*, a *CONSTANS-LIKE* gene, delays flowering by reducing expression of *CO* and *FT* in *Arabidopsis thaliana* . Plant J. 43, 758–768. doi: 10.1111/j.1365-313X.2005.02491.x 16115071

[B10] Chen H. Patterson N. Reich D. (2010). Population differentiation as a test for selective sweeps. Genome Res. 20, 393–402. doi: 10.1101/gr.100545.109 20086244 PMC2840981

[B11] Corbesier L. Vincent C. Jang S. Fornara F. Fan Q. Searle I.. (2007). FT protein movement contributes to long-distance signaling in floral induction of arabidopsis. Science 316, 1030–1033. doi: 10.1126/science.1141752 17446353

[B12] Cui X. Lu F. Li Y. Xue Y. Kang Y. Zhang S.. (2013). Ubiquitin-specific proteases UBP12 and UBP13 act in circadian clock and photoperiodic flowering regulation in arabidopsis. Plant Physiol. 162, 897–906. doi: 10.1104/pp.112.213009 23645632 PMC3668078

[B13] Cui Y. Xu Z. Xu Q. (2021). Elucidation of the relationship between yield and heading date using CRISPR/Cas9 system-induced mutation in the flowering pathway across a large latitudinal gradient. Mol. Breed. 41, 23. doi: 10.1007/s11032-021-01213-4 PMC10236111 37309418

[B14] Del Olmo I. Poza-Viejo L. Piñeiro M. Jarillo J. A. Crevillén P. (2019). High ambient temperature leads to reduced *FT* expression and delayed flowering in *Brassica rapa via* a mechanism associated with H2A.Z dynamics. Plant J. 100, 343–356. doi: 10.1111/tpj.14446 31257648

[B15] Ewels P. Magnusson M. Lundin S. Käller M. (2016). MultiQC: summarize analysis results for multiple tools and samples in a single report. Bioinformatics 32, 3047–3048. doi: 10.1093/bioinformatics/btw354 27312411 PMC5039924

[B16] Guo Y. Hans H. Christian J. Molina C. (2014). Mutations in single *FT*- and *TFL1*-paralogs of rapeseed (*Brassica napus* l.) and their impact on flowering time and yield components. Front. Plant Sci. 5. doi: 10.3389/fpls.2014.00282 PMC4060206 24987398

[B17] Guo T. Mu Q. Wang J. Vanous A. E. Onogi A. Iwata H.. (2020). Dynamic effects of interacting genes underlying rice flowering-time phenotypic plasticity and global adaptation. Genome Res. 30, 673–683. doi: 10.1101/gr.255703.119 32299830 PMC7263186

[B18] Han X. Xu Z. R. Zhou L. Han C. Y. Zhang Y. M. (2021). Identification of QTNs and their candidate genes for flowering time and plant height in soybean using multi-locus genome-wide association studies. Mol. Breed. 41, 39. doi: 10.1007/s11032-021-01230-3 PMC10236079 37309439

[B19] Helal M. M. U. Gill R. A. Tang M. Yang L. Hu M. Yang L.. (2021). SNP- and haplotype-based GWAS of flowering-related traits in *Brassica napus* . Plants 10, 2475. doi: 10.3390/plants10112475 34834840 PMC8619824

[B20] Hu J. Chen B. Zhao J. Zhang F. Xie T. Xu K.. (2022). Genomic selection and genetic architecture of agronomic traits during modern rapeseed breeding. Nat. Genet. 54, 694–704. doi: 10.1038/s41588-022-01055-6 35484301

[B21] Jiang D. Gu X. He Y. (2009). Establishment of the winter-annual growth habit *via FRIGIDA*-mediated histone methylation at *FLOWERING LOCUS c* in arabidopsis. Plant Cell 21, 1733–1746. doi: 10.1105/tpc.109.067967 19567704 PMC2714927

[B22] Kim D. H. Sung S. (2013). Coordination of the vernalization response through a *VIN3* and *FLC* gene family regulatory network in arabidopsis. Plant Cell 25, 454–469. doi: 10.1105/tpc.112.104760 23417034 PMC3608771

[B23] Knapp S. J. Stroup W. W. Ross W. M. (1985). Exact confidence intervals for heritability on a progeny mean basis. Crop Sci. 25, 192–194. doi: 10.2135/cropsci1985.0011183X002500010046x

[B24] Kusmec A. Srinivasan S. Nettleton D. Schnable P. S. (2017). Distinct genetic architectures for phenotype means and plasticities in zea mays. Nat. Plants 3, 715–723. doi: 10.1038/s41477-017-0007-7 29150689 PMC6209453

[B25] Kyung J. Jeon M. Jeong G. Shin Y. Seo E. Yu J.. (2022). The two clock proteins CCA1 and LHY activate *VIN3* transcription during vernalization through the vernalization-responsive cis-element. Plant Cell 34, 1020–1037. doi: 10.1093/plcell/koab304 34931682 PMC8894950

[B26] Li X. Guo T. Wang J. Bekele W. A. Sukumaran S. Vanous A. E.. (2021). An integrated framework reinstating the environmental dimension for GWAS and genomic selection in crops. Mol. Plant 14, 874–887. doi: 10.1016/j.molp.2021.03.010 33713844

[B27] Li Y. Huang Y. Bergelson J. Nordborg M. Borevitz J. O. (2010). Association mapping of local climate-sensitive quantitative trait loci in *Arabidopsis thaliana* . Proc. Natl. Acad. Sci. 107, 21199–21204. doi: 10.1073/pnas.1007431107 21078970 PMC3000268

[B28] Li M. Zhang Y. W. Xiang Y. Liu M. H. Zhang Y. M. (2022a). IIIVmrMLM: The r and c++ tools associated with 3VmrMLM, a comprehensive GWAS method for dissecting quantitative traits. Mol. Plant 15, 1251–1253. doi: 10.1016/j.molp.2022.06.002 35684963

[B29] Li M. Zhang Y. W. Zhang Z. C. Xiang Y. Liu M. H. Zhou Y. H.. (2022b). A compressed variance component mixed model for detecting QTNs, and QTN-by-environment and QTN-by-QTN interactions in genome-wide association studies. Mol. Plant 0. doi: 10.1016/j.molp.2022.02.012 35202864

[B30] Liu T. Carlsson J. Takeuchi T. Newton L. Farré E. M. (2013). Direct regulation of abiotic responses by the arabidopsis circadian clock component *PRR7* . Plant J. 76, 101–114. doi: 10.1111/tpj.12276 23808423

[B31] Liu J. Hua W. Yang H. L. Zhan G. M. Li R. J. Deng L. B.. (2012). The *BnGRF2* gene (*GRF2*-like gene from *Brassica napus*) enhances seed oil production through regulating cell number and plant photosynthesis. J. Exp. Bot. 63, 3727–3740. doi: 10.1093/jxb/ers066 22442419 PMC3388832

[B32] Liu J. Y. Zhang Y. W. Han X. Zuo J. F. Zhang Z. Shang H.. (2020). An evolutionary population structure model reveals pleiotropic effects of *GmPDAT* for traits related to seed size and oil content in soybean. J. Exp. Bot. 71, 6988–7002. doi: 10.1093/jxb/eraa426 32926130

[B33] Liu N. Du Y. Warbuton M. L. Xiao Y. Yan J. (2021). Phenotypic plasticity contributes to maize adaptation and heterosis. Mol. Biol. Evol. 38, 1262–1275. doi: 10.1093/molbev/msaa283 33212480 PMC8480182

[B34] López-González L. Mouriz A. Narro-Diego L. Bustos R. Martínez-Zapater J. M. Jarillo J. A.. (2014). Chromatin-dependent repression of the arabidopsis floral integrator genes involves plant specific PHD-containing proteins. Plant Cell 26, 3922–3938. doi: 10.1105/tpc.114.130781 25281686 PMC4247585

[B35] Love M. I. Huber W. Anders S. (2014). Moderated estimation of fold change and dispersion for RNA-seq data with DESeq2. Genome Biol. 15, 550. doi: 10.1186/s13059-014-0550-8 25516281 PMC4302049

[B36] Matar S. Kumar A. Holtgräwe D. Weisshaar B. Melzer S. (2021). The transition to flowering in winter rapeseed during vernalization. Plant Cell Environ. 44, 506–518. doi: 10.1111/pce.13946 33190312

[B37] Mouradov A. Cremer F. Coupland G. (2002). Control of flowering time: Interacting pathways as a basis for diversity. Plant Cell 14, S111–S130. doi: 10.1105/tpc.001362 12045273 PMC151251

[B38] Nakamichi N. Kita M. Niinuma K. Ito S. Yamashino T. Mizoguchi T.. (2007). Arabidopsis clock-associated pseudo-response regulators *PRR9*, *PRR7* and *PRR5* coordinately and positively regulate flowering time through the canonical *CONSTANS*-dependent photoperiodic pathway. Plant Cell Physiol. 48, 822–832. doi: 10.1093/pcp/pcm056 17504813

[B39] O’Neill C. M. Lu X. Calderwood A. Tudor E. H. Robinson P. Wells R.. (2019). Vernalization and floral transition in autumn drive winter annual life history in oilseed rape. Curr. Biol. 29, 4300–4306.e2. doi: 10.1016/j.cub.2019.10.051 31813609 PMC6926474

[B40] Patro R. Duggal G. Love M. I. Irizarry R. A. Kingsford C. (2017). Salmon provides fast and bias-aware quantification of transcript expression. Nat. Methods 14, 417–419. doi: 10.1038/nmeth.4197 28263959 PMC5600148

[B41] Putterill J. Laurie R. Macknight R. (2004). It’s time to flower: the genetic control of flowering time. BioEssays 26, 363–373. doi: 10.1002/bies.20021 15057934

[B42] Reeves P. H. Coupland G. (2000). Response of plant development to environment: control of flowering by daylength and temperature. Curr. Opin. Plant Biol. 3, 37–42. doi: 10.1016/S1369-5266(99)00041-2 10679453

[B43] Schiessl S. (2020). Regulation and subfunctionalization of flowering time genes in the allotetraploid oil crop *Brassica napus* . Front. Plant Sci. 11. doi: 10.3389/fpls.2020.605155 PMC7718018 33329678

[B44] Seo E. Lee H. Jeon J. Park H. Kim J. Noh Y. S.. (2009). Crosstalk between cold response and flowering in arabidopsis is mediated through the flowering-time gene *SOC1* and its upstream negative regulator *FLC* . Plant Cell 21, 3185–3197. doi: 10.1105/tpc.108.063883 19825833 PMC2782271

[B45] Shannon P. Markiel A. Ozier O. Baliga N. S. Wang J. T. Ramage D.. (2003). Cytoscape: A software environment for integrated models of biomolecular interaction networks. Genome Res. 13, 2498–2504. doi: 10.1101/gr.1239303 14597658 PMC403769

[B46] Sharma P. Mishra S. Burman N. Chatterjee M. Singh S. Pradhan A. K.. (2022). Characterization of *Cry2* genes (*CRY2a* and *CRY2b*) of *B. napus* and comparative analysis of *BnCRY1* and *BnCRY2a* in regulating seedling photomorphogenesis. Plant Mol. Biol. 110, 161–186. doi: 10.1007/s11103-022-01293-6 35831732

[B47] Shen Y. Xiang Y. Xu E. Ge X. Li Z. (2018). Major co-localized QTL for plant height, branch initiation height, stem diameter, and flowering time in an alien introgression derived *Brassica napus* DH population. Front. Plant Sci. 9. doi: 10.3389/fpls.2018.00390 PMC5883169 29643859

[B48] Siriwardana C. L. Gnesutta N. Kumimoto R. W. Jones D. S. Myers Z. A. Mantovani R.. (2016). NUCLEAR FACTOR y, subunit a (NF-YA) proteins positively regulate flowering and act through *FLOWERING LOCUS t* . PloS Genet. 12, e1006496. doi: 10.1371/journal.pgen.1006496 27977687 PMC5157953

[B49] Song J. Guan Z. Hu J. Guo C. Yang Z. Wang S.. (2020). Eight high-quality genomes reveal pan-genome architecture and ecotype differentiation of *Brassica napus* . Nat. Plants 6, 34–45. doi: 10.1038/s41477-019-0577-7 31932676 PMC6965005

[B50] Song J. Liu D. Xie W. Yang Z. Guo L. Liu K.. (2021). BnPIR: Brassica napus pan-genome information resource for 1689 accessions. Plant Biotechnol. J. 19, 412–414. doi: 10.1111/pbi.13491 33068485 PMC7955874

[B51] Tadege M. Sheldon C. C. Helliwell C. A. Stoutjesdijk P. Dennis E. S. Peacock W. J. (2001). Control of flowering time by *FLC* orthologues in *Brassica napus* . Plant J. 28, 545–553. doi: 10.1046/j.1365-313X.2001.01182.x 11849594

[B52] Tang S. Zhao H. Lu S. Yu L. Zhang G. Zhang Y.. (2021). Genome- and transcriptome-wide association studies provide insights into the genetic basis of natural variation of seed oil content in *Brassica napus* . Mol. Plant 14, 470–487. doi: 10.1016/j.molp.2020.12.003 33309900

[B53] Tan Z. Xie Z. Dai L. Zhang Y. Zhao H. Tang S.. (2022). Genome- and transcriptome-wide association studies reveal the genetic basis and the breeding history of seed glucosinolate content in *Brassica napus* . Plant Biotechnol. J. 20, 211–225. doi: 10.1111/pbi.13707 34525252 PMC8710833

[B54] Vidigal D. S. Marques A. C. S. S. Willems L. A. J. Buijs G. Méndez-Vigo B. Hilhorst H. W. M.. (2016). Altitudinal and climatic associations of seed dormancy and flowering traits evidence adaptation of annual life cycle timing in *Arabidopsis thaliana* . Plant Cell Environ. 39, 1737–1748. doi: 10.1111/pce.12734 26991665

[B55] Wang J. Long Y. Wu B. Liu J. Jiang C. Shi L.. (2009). The evolution of *Brassica napus FLOWERING LOCUS t* paralogues in the context of inverted chromosomal duplication blocks. BMC Evol. Biol. 9, 271. doi: 10.1186/1471-2148-9-271 19939256 PMC2794288

[B56] Wu R. (1998). The detection of plasticity genes in heterogeneous environments. Evolution 52, 967–977. doi: 10.1111/j.1558-5646.1998.tb01826.x 28565223

[B57] Xu J. Dai H. (2016). *Brassica napus cycling dof Factor1* (*BnCDF1*) is involved in flowering time and freezing tolerance. Plant Growth Regul. 80, 315–322. doi: 10.1007/s10725-016-0168-9

[B58] Xu L. Hu K. Zhang Z. Guan C. Chen S. Hua W.. (2016). Genome-wide association study reveals the genetic architecture of flowering time in rapeseed (*Brassica napus* l.). DNA Res. 23, 43–52. doi: 10.1093/dnares/dsv035 26659471 PMC4755526

[B59] Yang C. Gan Y. Harker K. N. Kutcher H. R. Gulden R. Irvine B.. (2014). Up to 32 % yield increase with optimized spatial patterns of canola plant establishment in western Canada. Agron. Sustain. Dev. 34, 793–801. doi: 10.1007/s13593-014-0218-5

[B60] Ying L. Chen H. Cai W. (2014). *BnNAC485* is involved in abiotic stress responses and flowering time in *Brassica napus* . Plant Physiol. Biochem. PPB 79, 77–87. doi: 10.1016/j.plaphy.2014.03.004 24690671

[B61] Yu H. Xu Y. Tan E. L. Kumar P. P. (2002). *AGAMOUS-LIKE 24*, a dosage-dependent mediator of the flowering signals. Proc. Natl. Acad. Sci. U. S. A. 99, 16336–16341. doi: 10.1073/pnas.212624599 12451184 PMC138612

[B62] Zhang H. Berger J. D. Milroy S. P. (2013a). Genotype×environment interaction studies highlight the role of phenology in specific adaptation of canola (*Brassica napus*) to contrasting Mediterranean climates. Field Crops Res. 144, 77–88. doi: 10.1016/j.fcr.2013.01.006

[B63] Zhang Y. Li B. Xu Y. Li H. Li S. Zhang D.. (2013b). The cyclophilin *CYP20-2* modulates the conformation of *BRASSINAZOLE*-*RESISTANT1*, which binds the promoter of *FLOWERING LOCUS d* to regulate flowering in arabidopsis. Plant Cell 25, 2504–2521. doi: 10.1105/tpc.113.110296 23897924 PMC3753379

[B64] Zhang J. Yi Q. Xing F. Tang C. Wang L. Ye W.. (2018). Rapid shifts of peak flowering phenology in 12 species under the effects of extreme climate events in Macao. Sci. Rep. 8, 13950. doi: 10.1038/s41598-018-32209-4 30224664 PMC6141562

[B65] Zhao H. Savin K. W. Li Y. Breen E. J. Maharjan P. Tibbits J. F.. (2022). Genome-wide association studies dissect the G × E interaction for agronomic traits in a worldwide collection of safflowers (*Carthamus tinctorius* l.). Mol. Breed. 42, 24. doi: 10.1007/s11032-022-01295-8 PMC10248593 37309464

[B66] Zhu Y. Cao Z. Xu F. Huang Y. Chen M. Guo W.. (2012). Analysis of gene expression profiles of two near-isogenic lines differing at a QTL region affecting oil content at high temperatures during seed maturation in oilseed rape (*Brassica napus* l.). Theor. Appl. Genet. 124, 515–531. doi: 10.1007/s00122-011-1725-2 22042481

[B67] Zou X. Suppanz I. Raman H. Hou J. Wang J. Long Y.. (2012). Comparative analysis of *FLC* homologues in brassicaceae provides insight into their role in the evolution of oilseed rape. PloS One 7, e45751. doi: 10.1371/journal.pone.0045751 23029223 PMC3459951

