# Supplementary material for: Genome-wide detection of genotype environment interactions for flowering time in Brassica napus
Source: Front Plant Sci. 2022 Nov 21;13:1065766. doi: 10.3389/fpls.2022.1065766 (PMC9721451; doi:10.3389/fpls.2022.1065766)
Supplement: Supplementary file 2 [file DataSheet_1.pdf]

Supplementary Figures

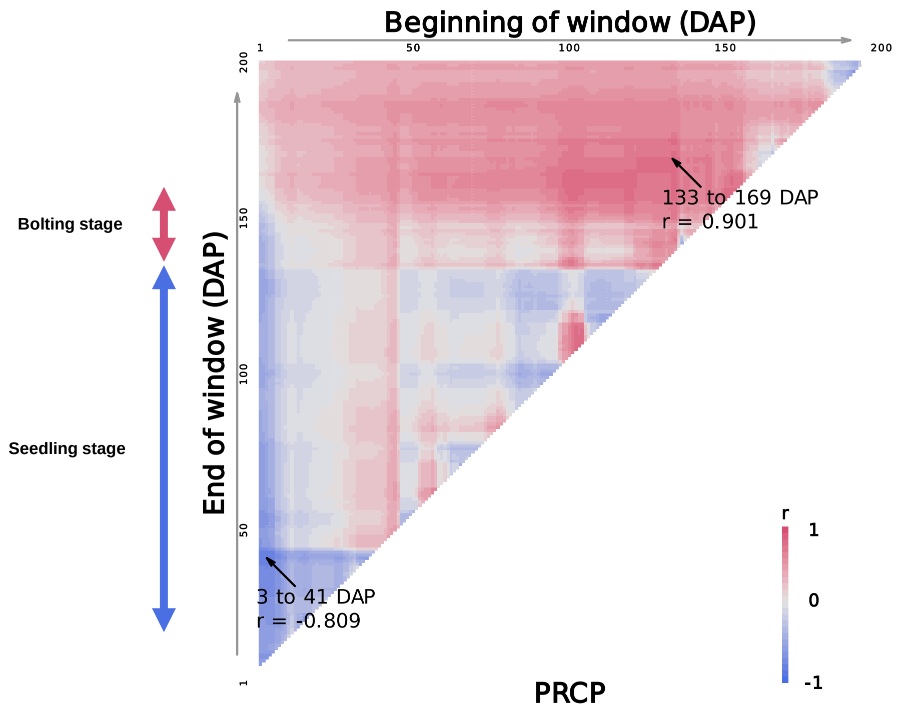

Supplementary Figure 1 Reaction norm of flowering time associated window to precipitation (PRCP). Search for the window to PRCP which is highly correlated with environmental mean of flowering time (from planting to 200 days after planting, DAP). PRCP within the window of 3-41 and 133-169 DAP was chosen and denoted as $PRCP_{3\text{-}41}$ and $PRCP_{133\text{-}169}$.

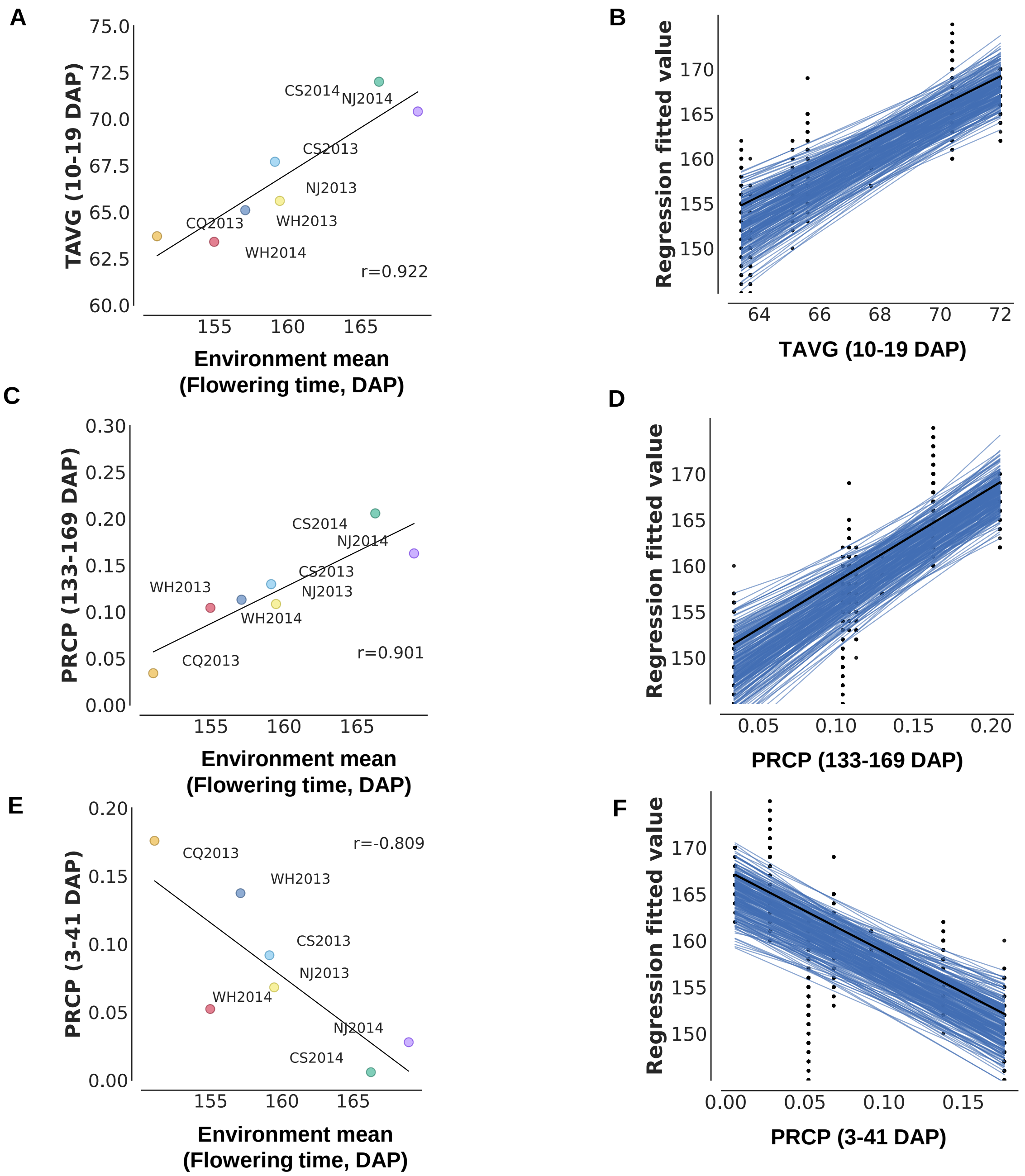

Supplementary Figure 2 Significant correlation **(A)** and reaction norm **(B)** between TAVG10-19 and environmental mean of flowering time. Significant correlation **(C)** and reaction norm (B) between $PRCP_{133\text{-}169}$ and environmental mean of flowering time. Significant correlation **(E)** and reaction norm **(F)** between $PRCP_{3\text{-}41}$ and environmental mean of flowering time.

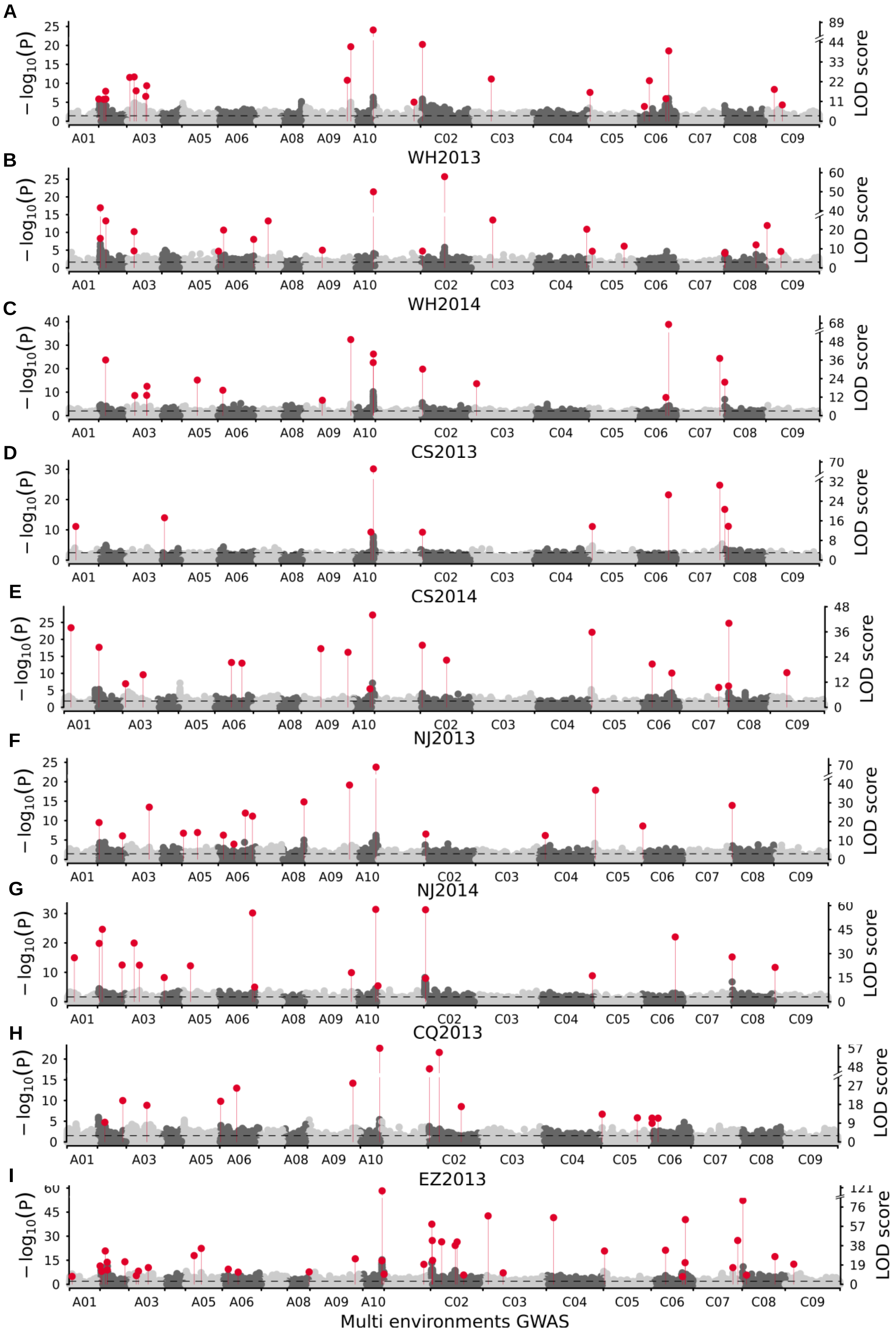

Supplementary Figure 3 Main-effect for flowering time in eight single environment analyses **(A-H)** and multiple environments joint analysis **(I)**. WH2013, Wuhan in 2013; WH2014, Wuhan in 2014; CS2013, Changsha in 2013; CS2014, Changsha in 2014; NJ2013, Nanjing in 2013; NJ2014, Nanjing in 2014; CQ2013, Chongqing in 2013; EZ2013, Ezhou in 2013.

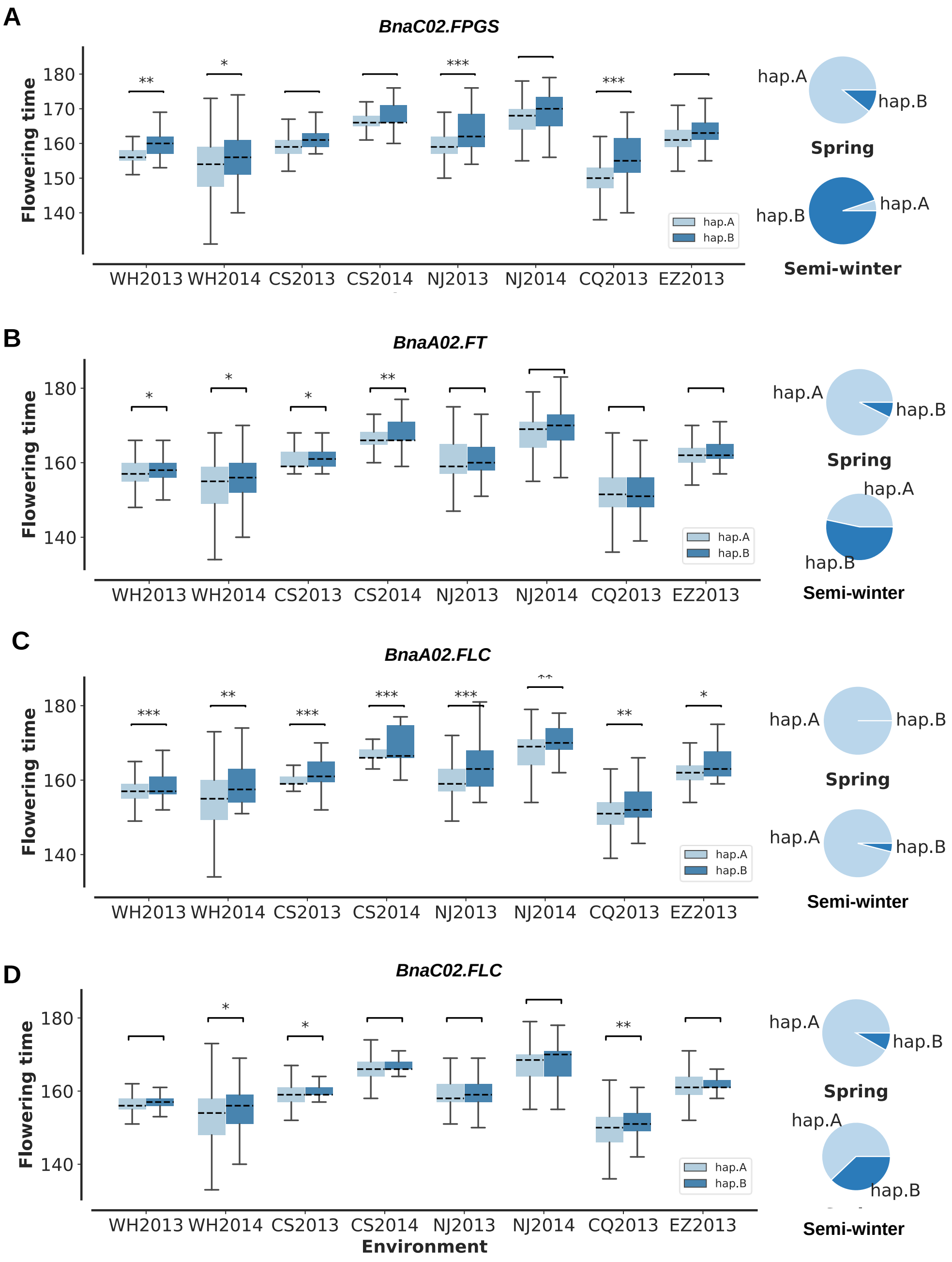

Supplementary Figure 4 Haplotype analysis of *BnaFPGS*, *BnaA02.FT*, *BnaA02.FLC*, and *BnaC02.FLC* **(A-D)**. In boxplot, significant differences for flowering time between each haplotype are calculated in eight environments with *t*-test. In pie plots, the haplotype frequencies of each gene in semi-winter and spring oilseed rapes are marked. WH2013, Wuhan in 2013; WH2014, Wuhan in 2014; CS2013, Changsha in 2013; CS2014, Changsha in 2014; NJ2013, Nanjing in 2013; NJ2014, Nanjing in 2014; CQ2013, Chongqing in 2013; EZ2013, Ezhou in 2013. *P = 0.05, **P = 0.01, and ***P = 0.001.
